# Glycyrrhizin Attenuates *Salmonella* Typhimurium-Induced Tissue Injury, Inflammatory Response, and Intestinal Dysbiosis in C57BL/6 Mice

**DOI:** 10.3389/fvets.2021.648698

**Published:** 2021-06-22

**Authors:** Baikui Wang, Xiaolin Ye, Yuanhao Zhou, Pengwei Zhao, Yulong Mao

**Affiliations:** ^1^Key Laboratory of Animal Molecular Nutrition of Education of Ministry, National Engineering Laboratory of Biological Feed Safety and Pollution Prevention and Control, Key Laboratory of Animal Feed and Nutrition of Zhejiang Province, Institute of Animal Nutrition and Feed Sciences, College of Animal Sciences, Zhejiang University, Hangzhou, China; ^2^Medical Faculty, Heinrich-Heine University Düsseldorf, Düsseldorf, Germany

**Keywords:** glycyrrhizin, *Salmonella* Typhimurium, tissue injury, inflammatory response, intestinal dysbiosis

## Abstract

*Salmonellae* are one of the most important foodborne pathogens, which threaten the health of humans and animals severely. Glycyrrhizin (GL) has been proven to exhibit anti-inflammatory and tissue-protective properties. Here, we investigated the effects of GL on tissue injury, inflammatory response, and intestinal dysbiosis in *Salmonella* Typhimurium-infected mice. Results showed that GL or gentamicin (GM) significantly (*P* < 0.05) alleviated ST-induced splenomegaly indicated by the decreased spleen index, injury of liver and jejunum indicated by the decreased hepatocytic apoptosis, and the increased jejunal villous height. GL significantly (*P* < 0.05) increased secretion of inflammatory cytokines (IFN-γ, IL-12p70, IL-6, and IL-10) in spleen and IL-12p40 mRNA expression in liver. Meanwhile, GL or GM pre-infection treatments significantly (*P* < 0.05) decreased ST-induced pro-inflammatory cytokine (IFN-γ, TNF-α, and IL-6) expression in both spleen and liver and increased (*P* < 0.05) anti-inflammatory cytokine IL-10 secretion in spleen. Furthermore, GL or GM pre-infection treatment also regulates the diversities and compositions of intestinal microbiota and decreased the negative connection among the intestinal microbes in ST-infected mice. The above findings indicate that GL alleviates ST-induced splenomegaly, hepatocytic apoptosis, injury of jejunum and liver, inflammatory response of liver and spleen, and intestinal dysbacteriosis in mice.

## Introduction

*Salmonellae* are facultative intracellular bacterial pathogens, which can invade and survive inside many cell types, such as epithelial cells, macrophages, microfold (M) cells, and dendritic cells (1, 2). As one of the most common foodborne pathogens, *Salmonellae* had strong pathogenicity, which colonizes, adheres to, and damages the intestinal epithelium by producing enterotoxins and then invade organs (such as liver, spleen, and kidney) by secreting invasive protein to induce organic swellings and inflammation (3, 4). *Salmonellae*-induced *Salmonellosis* poses a great threat to the health of food animals and humans. It is reported that *Salmonellae*-contaminated food-induced *Salmonellosis* is one of the major causes of diarrhea of humans globally (5), and the total economic loss caused by *Salmonellosis* is estimated to be over $3.5 billion per year in the US (6). *Salmonellae* infection not only causes enteric diseases that compromise growth performance and overall health of animals and thereby lead to serious economic loss for food animal industry but also leads to serious *Salmonellae*-contaminated raw food animal products (meat and eggs) (6, 7). *Salmonellae*-contaminated poultry products (raw meat and eggs) are considered as the leading food sources for human *Salmonellosis* (8). Traditionally, in-feed antibiotics is the main strategy to prevent or control *Salmonellae*-induced enteric diseases in animal production (9). As growth promoters, antibiotics have been widely used in livestock production since the 1940's to improve growth performance and overall health of animals (10). However, with the increasing concerns about antimicrobial resistance (AMR) and foodborne antibiotic residues, many countries, including China, have banned the use of antibiotics in animal husbandry production (11–13). With the strict ban of the in-feed antibiotic growth promoters (AGPs), gastrointestinal infectious diseases of food animals and zoonotic pathogen contamination in animal products severely threaten the health of animals and humans (11, 14). Therefore, it is a priority to explore the proper alternatives to AGP under the post-AGP era (13). In recent years, many studies have been reported that some natural agents, such as natural plant extracts, probiotics, and prebiotics, are beneficial for improving growth performance and reducing morbidity and mortality of food animals, and are considered “Generally Recognized as Safe (GRAS)” alternatives to AGP (13, 15–17).

Glycyrrhizin (GL), a triterpene glycoside that is extracted from licorice root, consists of one molecule 18-β-glycyrrhetinic acid and two molecules of glucuronic acid and is the most important active ingredient of licorice root (18, 19). GL has been proven to exert a variety of pharmacological activities, such as anti-microbial, anti-inflammatory, anti-viral, anti-oxidative, anti-tumor, and hepatoprotective activities (18, 20–22). GL can activate specific and non-specific immune responses by enhancing phagocytosis and bactericidal activities of macrophage (18), inducing maturation of dendritic cells (DCs) and proliferation of T lymphocytes (23), augmenting natural killer (NK) cell activity (24), and inducing cytokine secretion (18). GL exhibits anti-inflammatory and tissue-protective properties by binding to high-mobility group box (HMGB1) to inhibit cytokine secretion activities (25). Many studies showed that as an alternative to antibiotics, GL had a beneficial effect in preventing or controlling multi-drug-resistant pathogen infection (26, 27). As a potential substitute for AGP, licorice extract showed beneficial effects on the growth performance of broiler (28, 29). Our previous studies also found that GL exerts anti-*Salmonella* activities by inducing M1 polarization of murine bone marrow-derived macrophages (BMDMs) and maturation of murine bone marrow-derived dendritic cells (BMDCs) (18, 30). The present study was aimed to further investigate whether GL as a potential AGP substitute has protective effects against ST-induced tissue injury, inflammatory response, and intestinal dysbiosis in C57BL/6 mice.

## Materials and Methods

### Reagents

GL was purchased from Sigma-Aldrich (purity ≥95.0%, St. Louis, MO, USA). Gentamicin (GM) was purchased from Sigma-Aldrich (St. Louis, MO, USA). ELISA kits for IFN-γ, IL-12p70, TNF-α, IL-6, and IL-10 were obtained from eBioscience (San Diego, CA, USA). Caspase-1 activity assay kit was purchased from Beyotime Institute of Biotechnology (Shanghai, China).

### Bacteria Preparation

ST CMCC 50115 was generously provided by Dr. Weihuan Fang (Institute of Preventive Veterinary Medicine, Zhejiang University). ST was cultured at 37°C in Luria–Bertani broth overnight under aerobic conditions. The ST pellet was harvested by centrifugation at 4000 × *g* for 15 min at 16°C and then washed three times with sterile phosphate-buffered saline (pH 7.2). Finally, the optical density method (SpectraMax M5, MD, USA) and spreading plate method were conducted to adjust the final ST.

### Animal Experimental Design

Male C57BL/6 mice (6 weeks old, Slac Animal Inc., Shanghai, China) were raised in the Experimental Animal Center of Zhejiang University under light-controlled (12-h light/dark cycle) and temperature-controlled (22 ± 1°C) conditions and had free access to water and food. Fifty male C57BL/6 mice were randomly divided into five groups (*n* = 10/group): Control group, glycyrrhizin-treated group (GL), ST-infected group (ST), glycyrrhizin protective group (GL + ST), and gentamicin protective group (GM + ST, as a positive protective group). Mice in the Control and ST groups were drinking sterile water. The mice in the GL, GL + ST, and GM + ST groups were drinking sterile water containing 0.4 mg/ml GL (80 mg/kg weight) or 0.4 mg/ml GM (80 mg/kg weight) every day, respectively (30). All mice were fed a basal diet and weighted every 3 days. After 3 weeks, mice were orally infected with 200 μl of *Salmonella* Typhimurium (2 × 10^9^ CFU/ml) (31). Mice were euthanized at day 3 post-infection. Spleen was weighted, and liver, jejunum, and cecum were collected for further analysis. Spleen index (*n* = 10/group) was calculated according to the formula: Spleen index = spleen weight (mg)/body weight (g).

### Hematoxylin and Eosin Staining and TUNEL Assay

For photonic microscope observations, the liver and jejunal samples (*n* = 5/group) of mice were fixed in 4% paraformaldehyde, embedded in paraffin, sliced, dehydrated with gradient concentrations of alcohol, and then stained with hematoxylin and eosin (H&E). Images were captured, and the villus height was measured by an Olympus microsystem (Tokyo, Japan). The TUNEL assay was determined by using TUNEL Assay Kit (Abcam, Cambridge, United States) according to the manufacturer's instructions. Briefly, the paraffin-embedded liver sections were deparaffinized with xylene, hydrated with gradient concentrations of alcohol, and covered with proteinase K. Slides were incubated with terminal deoxynucleotidyl transferase and biotinylated nucleotides and then treated with saline-sodium citrate buffer, 6% hydrogen peroxide, streptavidin–HRP conjugate, and DAB substrate solution. Finally, the slides were counterstained in hematoxylin solution. Images were captured using an Olympus microsystem (Tokyo, Japan) and the apoptotic cells were quantified using ImageJ analysis software (National Institute of Mental Health, Maryland, USA). Apoptosis index was calculated according to the formula: Apoptosis index (%) = [the number of apoptotic cells/(the number of apoptotic cells + the number of intact cells)] ×100.

### ELISA and Caspase-1 Activity Analyses

The spleen and liver samples (*n* = 5/group) were homogenized with ice-cold sterile saline solution (1:9, w/v) and centrifuged at 4000 × *g* for 20 min at 4°C. Then, the collected supernatant was used for ELISA and caspase-1 activity analyses. Levels of interferon-γ (IFN-γ), interleukin-12 subunit p70 (IL-12p70), tumor necrosis factor-α (TNF-α), IL-6, and IL-10 in the spleen homogenates were colorimetrically determined by enzyme-linked immunosorbent assay (ELISA) kits (eBioscience, San Diego, CA) according to the manufacturer's instructions. Caspase-1 activity in liver homogenates were determined by caspase-1 activity assay kit according to the manufacturer's instructions.

### RT-qPCR Analysis

Total RNA (*n* = 5/group) was extracted from spleen tissue using RNAiso Plus kit (TAKARA, Dalian, China). Reverse transcription was performed using the PrimeScript II 1st Strand cDNA Synthesis Kit (TAKARA) according to the manufacturer's recommendation. RT-qPCR was conducted using SYBR PremixEx TaqII (TAKARA) by the StepOne real-time PCR system (Applied Biosystems). All primer sequences for target genes (including TNF-α, IL-12p40, IL-6, IFN-γ, and IL-10) are listed in Table 1. Fold changes were calculated after normalizing to the housekeeping gene β-actin using the 2^−ΔΔCt^ method.

**Table 1:** Primer sequences used for qRT-PCR.

**Gene name**	**Primer sequence (5^**′**^-3^**′**^)**	**Product size**	**Accession no**.
TNF-α	F: CCCTCACACTCAGATCATCTTCT	61	NM_013693
	R: GCTACGACGTGGGCTACAG		
IL-12 p40	F: CCCATTCCTACTTCTCCCTCAA	75	NM_001303244
	R: CCTCCTCTGTCTCCTTCATCTT		
IL-6	F: TAGTCCTTCCTACCCCAATTTCC	76	NM_031168
	R: TTGGTCCTTAGCCACTCCTTC		
IFN-γ	F: TCAGCAACAACATAAGCGTCAT	104	NM_008337
	R: GACCTCAAACTTGGCAATACTCA		
IL-10	F: GCTCTTACTGACTGGCATGAG	105	NM_010548
	R: CGCAGCTCTAGGAGCATGTG		
β-actin	F: CGTTGACATCCGTAAAGACC	281	NM_007393
	R: AACAGTCCGCCTAGAAGCAC		

*TNF-α, tumor necrosis factor alpha; IL, interleukin; IFN-γ, interferon gamma; F, forward; R, reverse*.

### Microbial Analysis

The cecal bacterial genomic DNA (*n* = 4/group) was extracted using the TIANamp Stool DNA Kit (Tiangen, Beijing, China) according to the manufacturer's instructions, and the quality of extracted DNA was checked by agarose gel electrophoresis and spectrophotometric analysis. The V3–V4 region of the 16S rRNA gene was amplified using the primer pair 341F/805R, and sequencing was performed on MiSeq platform (Illumina Inc., San Diego, CA, USA). Sequences were filtered and clustered into operational taxonomic unit (OTU) at 97% similarity by QIIME software (version 1.9.1). Bacterial OTU representative sequences were assigned to a taxonomic lineage by Ribosomal Database Project (RDP) classifier based on the SILVA database (SILVA 132 release).

Alpha diversity (observed OTUs and PD_whole_tree) and beta diversity were analyzed based on a subsample of a minimum number of sequences (12,722) by QIIME software. Beta diversity was displayed by principal coordinates analysis (PCoA) using R software (https://www.r-project.org/). Analysis of similarities (ANOSIM), permutational multivariate analysis of variance (PERMANOVA), and multi response permutation procedure (MRPP) were calculated using “vegan” package of R software to determine significant differences in bacterial beta diversity among the five groups (based on the Bray–Curtis distance matrices) (32).

### Statistical Analysis

Pearson correlation between phenotypic variables and the relative abundance of microbial communities (phylum level) were analyzed and visualized by the package “corrplot” of R software. The co-occurrence networks of microbial communities in different treatments were built based on significant correlations (Spearman's *R* > 0.6 and FDR-adjusted *P* < 0.05) (33) and were visualized by Gephi™ software (https://gephi.org/). The topological properties of the co-occurrence network were also calculated to describe the complex patterns of the interrelationships by Gephi software. Comparison of the intestinal bacteria among different treatments was analyzed and visualized by statistical analysis of taxonomic and functional profiles (STAMP) with a 95% confidence interval. The rest of the data were evaluated by ANOVA, and the contrast of means was performed by Tukey's multiple range test, using SPSS™ software (SPSS Inc., Chicago, IL, USA), and the graphs were generated using Origin 8.5™ (OriginLab, Berkeley, CA, USA). The statistical significance was set at *P* < 0.05.

## Results

### GL Attenuates ST-Induced Jejunum Injury

**Figure 1:**
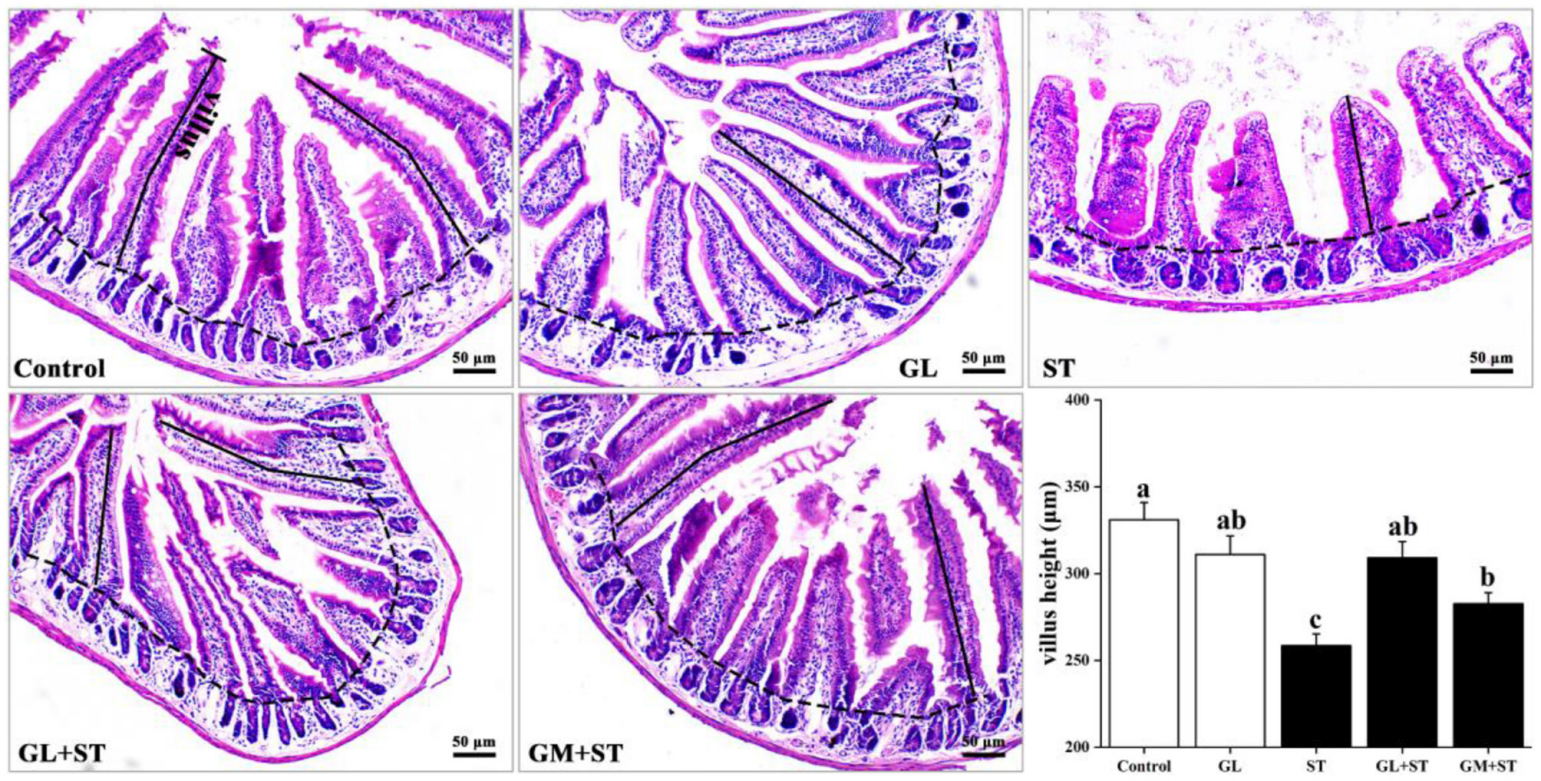
Effect of GL on jejunum morphology in ST-infected mice. Representative images of the jejunum stained with H&E. Results are presented as mean ± SD (*n* = 5/group). Different lowercase letters indicate a significant difference (*P* < 0.05).

H&E staining showed that compared with the Control group, GL treatment had little effect on villous height (*P* > 0.05) and jejunal structure, which exhibited integrated structure, ordered jejunal villi, and completed gland (Figure 1). However, compared with the Control group, ST infection significantly (*P* < 0.05) decreased the villous height of jejunum. Compared with the ST group, GL or GM significantly (*P* < 0.05) prevented jejunum injury indicated by the higher villous height (Figure 1).

### GL Alleviates ST-Induced Splenomegaly

**Figure 2:**
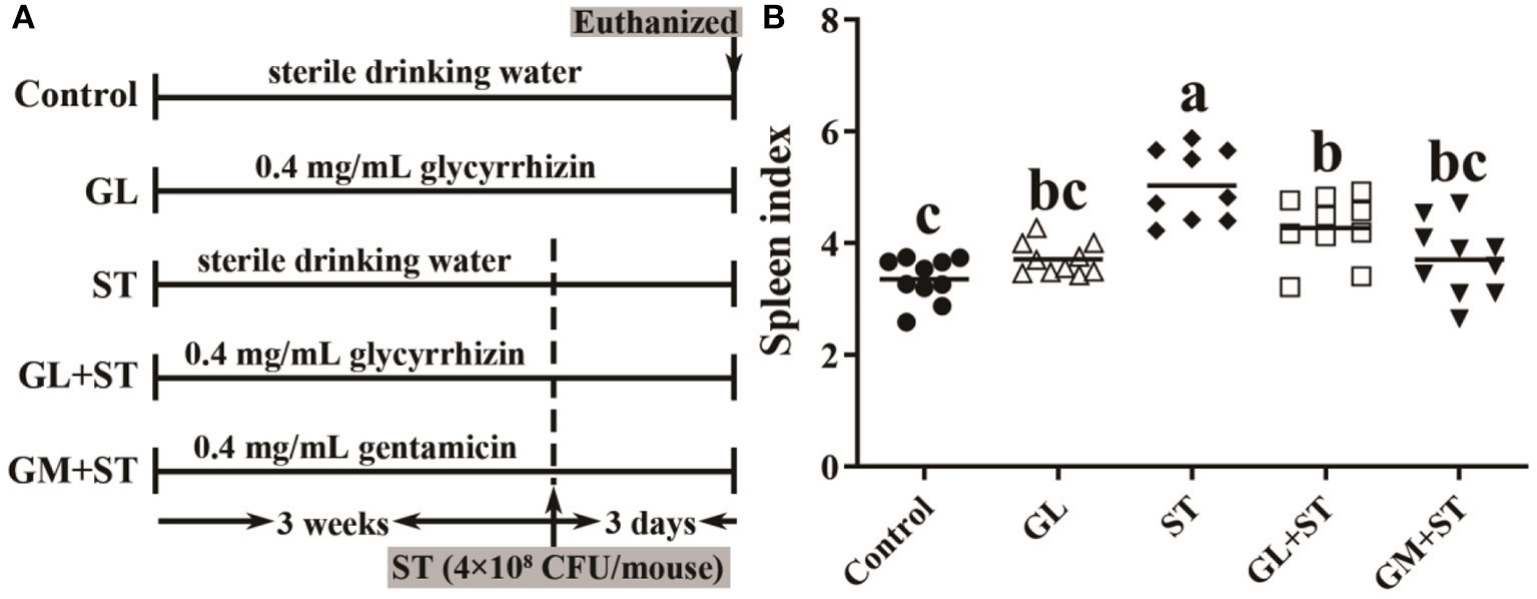
GL alleviates ST-induced splenomegaly. **(A)** Graphical outline of the animal study. **(B)** Spleen index. Results are presented as mean ± SD (*n* = 10/group). Different lowercase letters indicate a significant difference (*P* < 0.05).

As shown in Figure 2, compared with the Control group, the spleen index of mice was significantly (*P* < 0.05) increased in the ST-infected group by 50.18%. GL or GM pretreatments significantly (*P* < 0.05) attenuated ST-induced splenomegaly by 15.16 or 26.35%, while there was no significant difference between the two pretreated groups (*P* > 0.05).

### GL Attenuates ST-Induced Liver Injury

**Figure 3:**
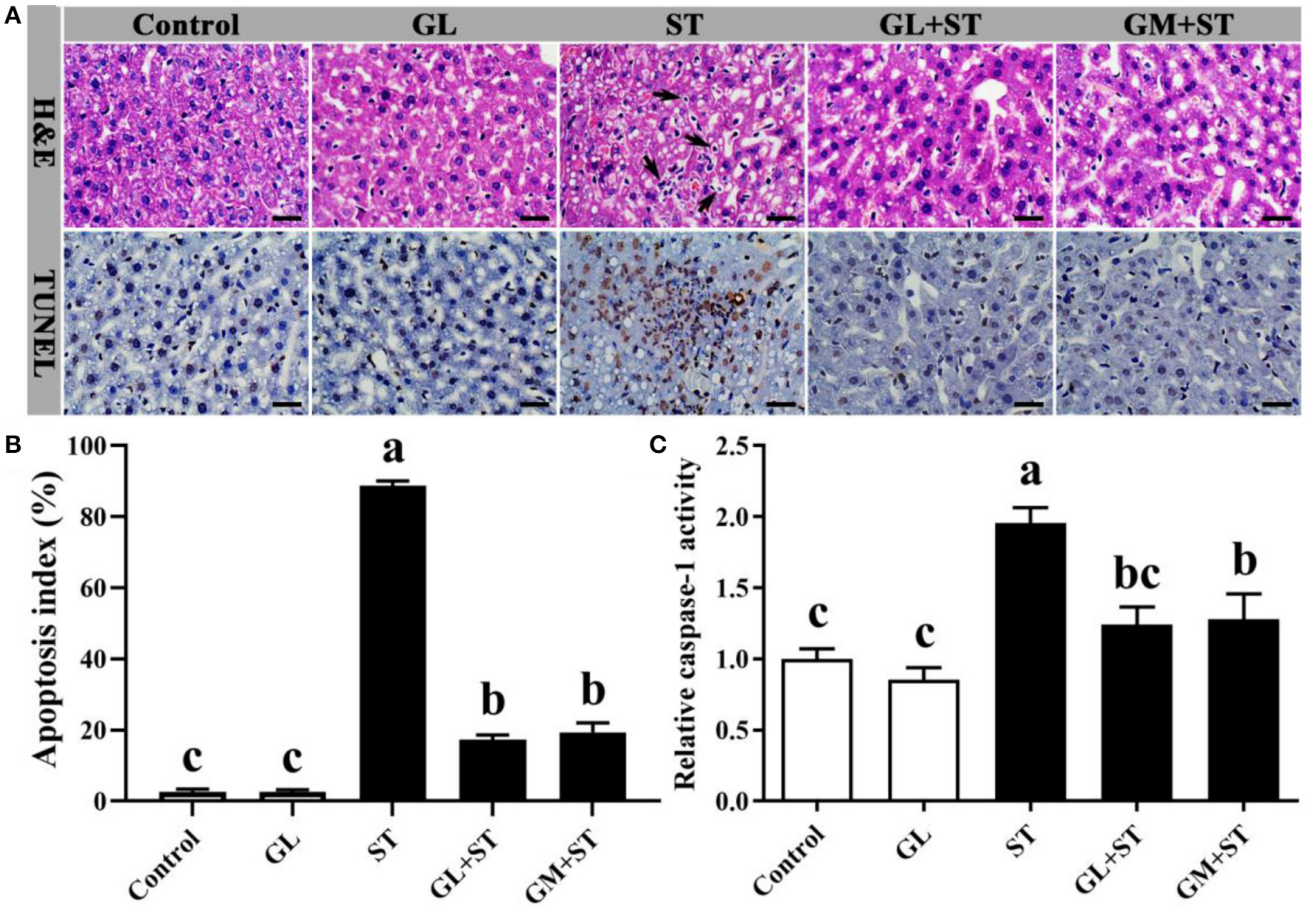
Effect of GL on liver morphology and hepatocytic apoptosis in ST-infected mice. **(A)** H&E staining and TUNEL assay (scale bar, 50 μm). H&E: Black arrow indicates apoptotic cells. TUNEL assay: Apoptotic cells were labeled as brown, and intact cells were labeled as blue. **(B)** Apoptosis index was calculated according to the formula: Apoptosis index (%) = [the number of apoptotic cells/(the number of apoptotic cells + the number of intact cells)] × 100. **(C)** Caspase-1 activity in liver was determined by caspase-1 activity assay kit. Results are presented as mean ± SD (*n* = 5/group). Different lowercase letters indicate a significant difference (*P* < 0.05).

Compared with the Control group, GL treatment did not cause hepatocytic apoptosis exhibiting completed structure, hepatic lobule, and intact cell and unincreased apoptosis index (*P* > 0.05) (Figures 3A,B). However, ST infection caused hepatocytes with fragmented and pyknotic nuclei (Figure 3A, H&E, black arrows indicted) and hepatocytic apoptosis (Figure 3A, TUNEL, cells labeled as brown) and significantly (*P* < 0.05) increased apoptosis index (Figure 3B), which could be significantly (*P* < 0.05) alleviated by GL or GM pretreatments (Figures 3A,B). Compared with the Control group, GL treatment had no effect on caspase-1 activity (*P* > 0.05), while ST infection significantly (*P* < 0.05) increased the activity of caspase-1, which could be significantly (*P* < 0.05) reversed by GL or GM pretreatments (Figure 3C).

### GL Alleviates ST-Induced Spleen and Liver Inflammation

**Figure 4:**
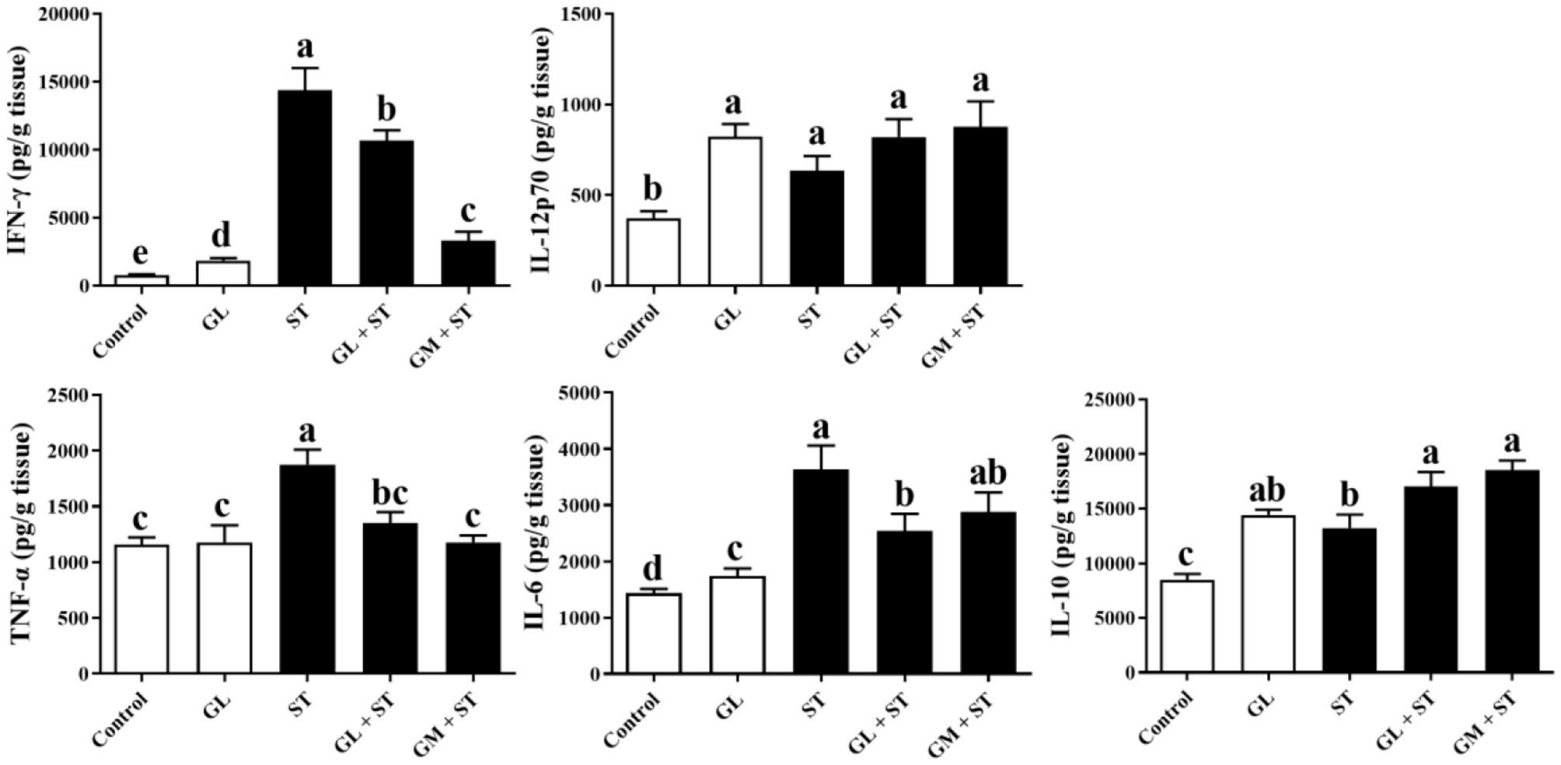
Effect of GL on splenic cytokine production in ST-infected mice. The levels of pro-inflammatory cytokine (IFN-γ, IL-12p70, TNF-α, and IL-6) and anti-inflammatory cytokine (IL-10) in spleen were determined by ELISA kit. Results are presented as mean ± SD (*n* = 5/group). Different lowercase letters indicate a significant difference (*P* < 0.05).

As shown in Figure 4, compared with the Control group, GL treatment significantly (*P* < 0.05) increased the secretions of IFN-γ, IL-12p70, IL-6, and IL-10 in spleen, whereas, it had no (*P* > 0.05) effect on the protein level of TNF-α. ST infection significantly (*P* < 0.05) increased the secretion of pro-inflammatory cytokines (IFN-γ, IL-12p70, TNF-α, and IL-6) and anti-inflammatory cytokine (IL-10) in spleen. As expected, GL or GM pretreatments effectively (*P* < 0.05) decreased the increased pro-inflammatory cytokines (IFN-γ, TNF-α, and IL-6) induced by ST infection, whereas, they increased anti-inflammatory cytokine IL-10 secretion (*P* < 0.05).

**Figure 5:**
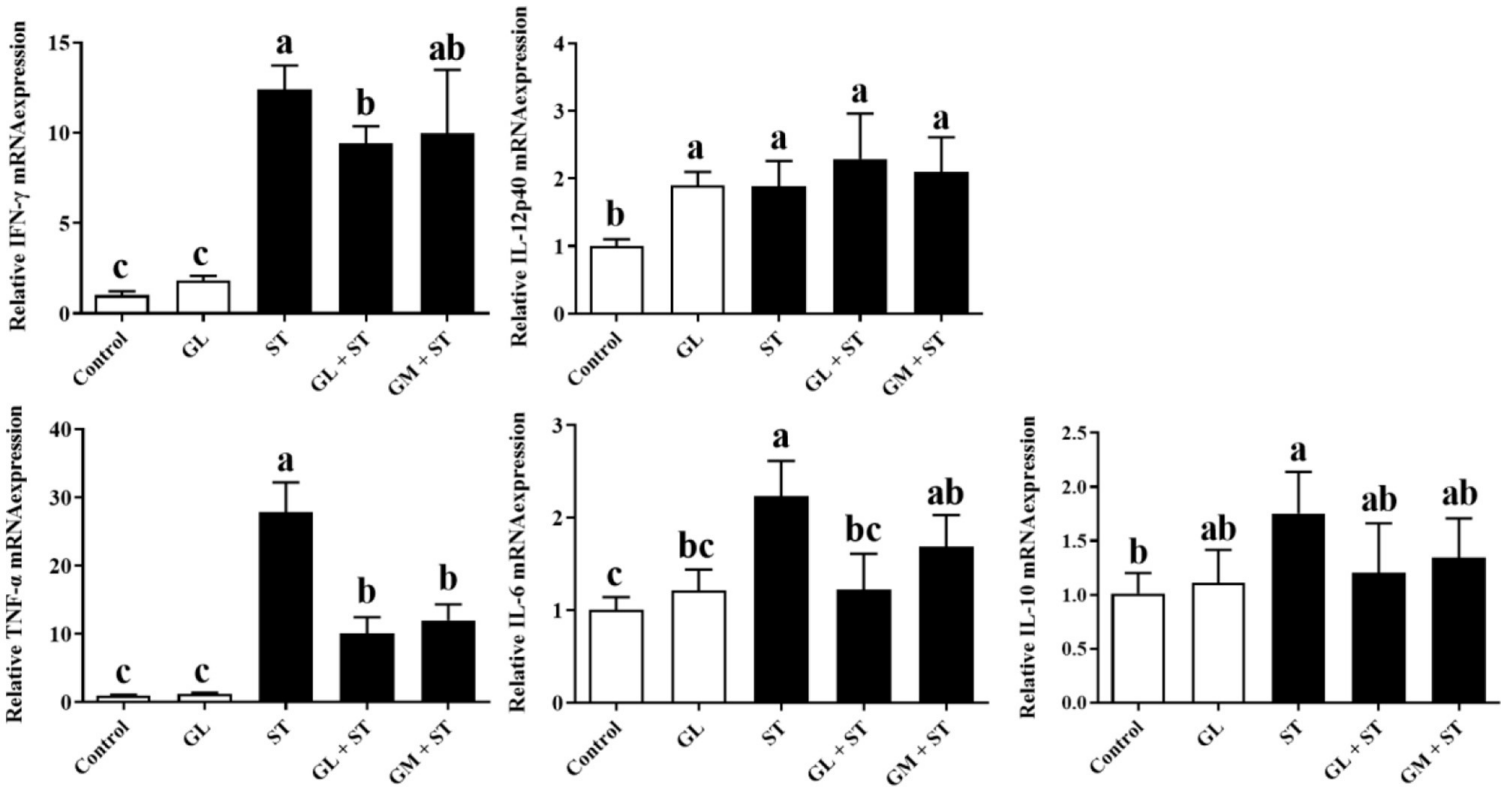
Effect of GL on mRNA expression of liver cytokines in ST-infected mice. The expression of pro-inflammatory cytokine (IFN-γ, IL-12p40, TNF-α, and IL-6) and anti-inflammatory cytokine (IL-10) in liver was measured by RT-qPCR. Results are presented as mean ± SD (*n* = 5/group). Different lowercase letters indicate a significant difference (*P* < 0.05).

Furthermore, the mRNA expression of cytokines in liver showed that compared with the Control group, GL treatment significantly (*P* < 0.05) upregulated the IL-12p40 mRNA expression, whereas, it had no significant (*P* > 0.05) effect on the expression of IFN-γ, TNF-α, IL-6, and IL-10 (Figure 5). ST infection significantly (*P* < 0.05) increased the mRNA expression of pro-inflammatory cytokines (IFN-γ, IL-12p40, TNF-α, and IL-6) and anti-inflammatory cytokine (IL-10). Similarly, GL or GM pretreatments also significantly (*P* < 0.05) decreased the mRNA expression of IFN-γ, TNF-α, and IL-6 (Figure 5).

### GL Modulates ST-Induced Intestinal Dysbiosis

**Figure 6:**
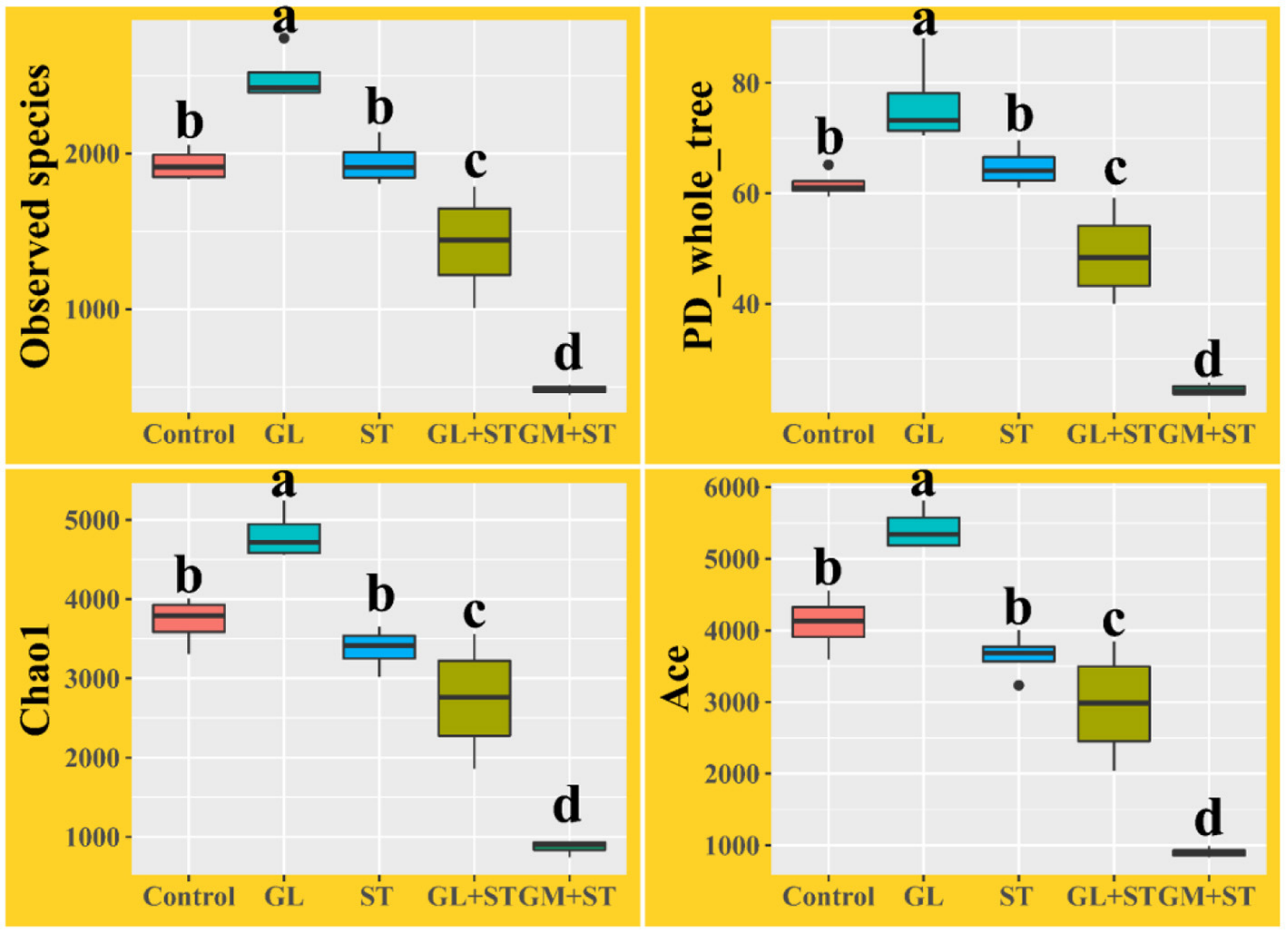
Alpha diversity analysis of intestinal microbiota in different groups (*n* = 4/group). Different lowercase letters indicate a significant difference (*P* < 0.05).

**Figure 7:**
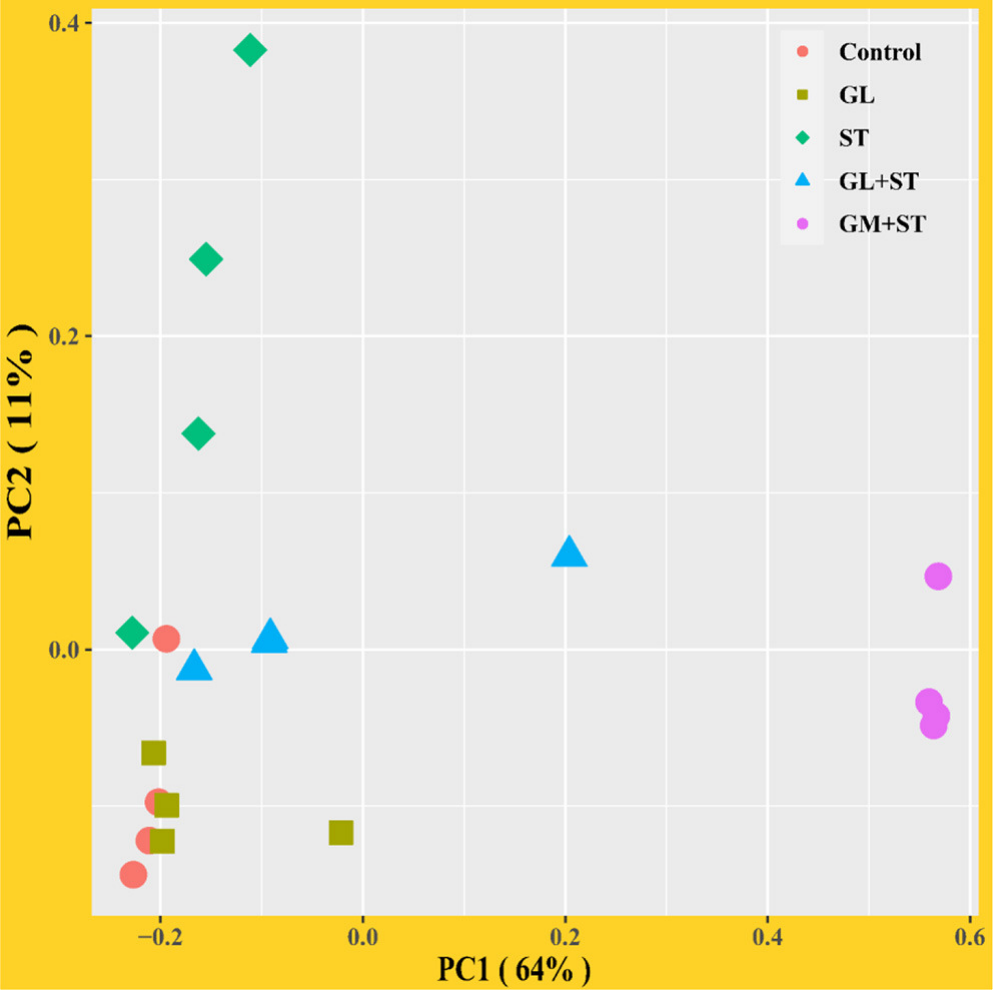
Principal coordinates analysis (PCoA) of microbial communities among groups based on Bray–Curtis distance (*n* = 4/group).

Alpha diversity analysis showed that compared with the Control group, GL treatment significantly (*P* < 0.05) increased the alpha diversities (including observed OTUs, PD_whole_tree index, Chao1, and Ace) of the intestinal microbiota, while ST infection had no (*P* > 0.05) effect on the alpha diversities (Figure 6). Additionally, the alpha diversity indices of the microbial communities in the GL + ST and GM + ST treatment were significantly (*P* < 0.05) lower than those of the ST group. PCoA of microbial communities based on Bray–Curtis distance suggested variation of the bacterial community structure with treatments (Figure 7). Significant differences in beta diversity among treatments were further confirmed by ANOSIM, PERMANOVA, and MRPP analysis (Table 2), except for that between the Control and GL treatments.

**Table 2:** ANOSIM, PERMANOVA, and MRPP analysis of microbial diversity among different treatments (*n* = 4/group).

	**ANOSIM**		**ADONIS**		**MRPP**	
	***R***	***P***	***R*^**2**^**	***P***	***A***	***P***
Treatment	0.653	0.001	0.765	0.001	0.451	0.001
Control vs. GL	0.000	0.586	0.202	0.129	0.034	0.181
Control vs. ST	0.688	0.028	0.457	0.045	0.204	0.034
ST vs. GL + ST	0.313	0.071	0.327	0.023	0.111	0.029
ST vs. GM + ST	1.000	0.032	0.848	0.043	0.577	0.023
GL + ST vs. GM + ST	1.000	0.028	0.722	0.024	0.450	0.021

**Figure 8:**
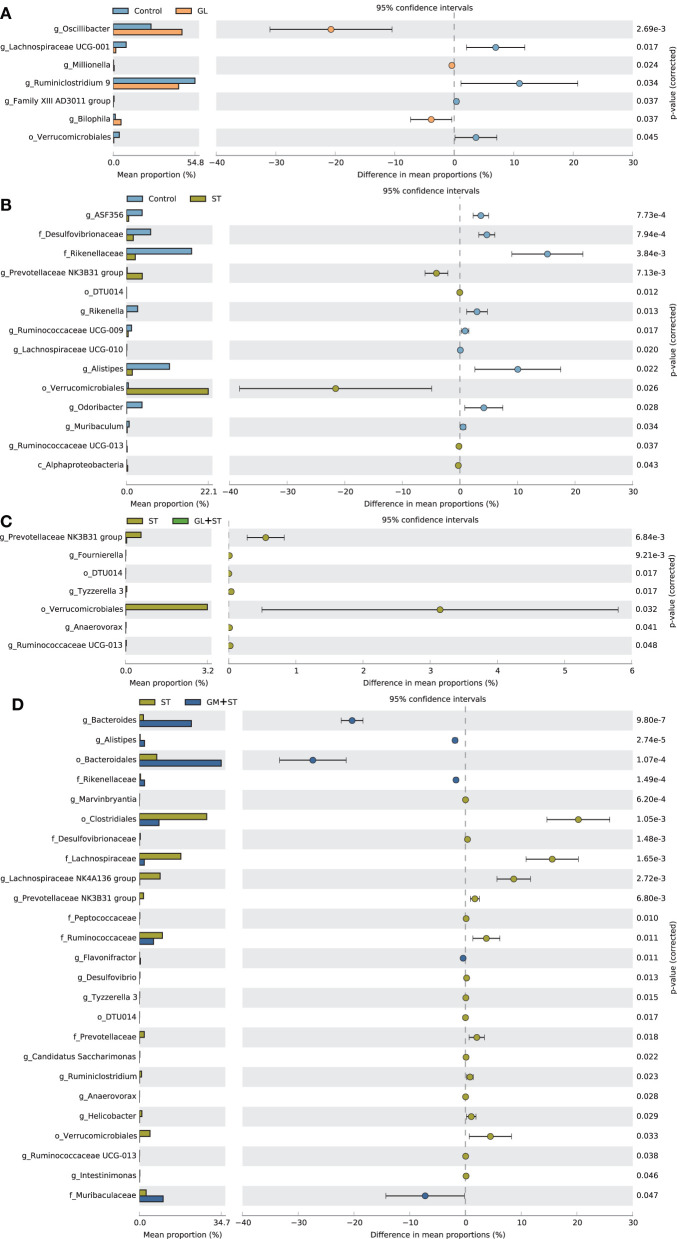
Comparison of the intestinal bacteria among different treatments. **(A)** Control vs. GL, **(B)** Control vs. ST, **(C)** ST vs. GL + ST, **(D)** Control vs. GM + ST. Confidence Interval was set at 95%.

The differences of the intestinal bacterial compositions among groups were also analyzed (Figure 8). The results showed that GL treatment significantly (*P* < 0.05) increased the relative abundance of *Oscillibacter, Millionella*, and *Bilophila*, whereas, it decreased (*P* < 0.05) the relative abundance of *Verrucomicrobiales*, Family XIII AD3011 group, *Ruminiclostridium* 9, and *Lachnospiraceae* UCG-001. ST infection significantly (*P* < 0.05) increased the relative abundance of *Alphaproteobacteria, Verrucomicrobiales*, DTU014, *Prevotellaceae* NK3B31 group, and *Ruminococcaceae* UCG-013, whereas, it decreased (*P* < 0.05) the relative abundance of *Desulfovibrionaceae, Rikenellaceae*, ASF356, *Rikenella, Ruminococcaceae* UCG-009, *Lachnospiraceae* UCG-010, *Alistipes, Odoribacter*, and *Muribaculum*. Moreover, GL or GM pretreatments significantly (*P* < 0.05) decreased ST-induced relative abundance of *Verrucomicrobiales*, DTU014, *Prevotellaceae* NK3B31 group, and *Ruminococcaceae* UCG-013. The relative abundances of *Rikenellaceae* and *Alistipes* were significantly (*P* < 0.05) increased in the GM + ST group compared with those in the ST group.

**Figure 9:**
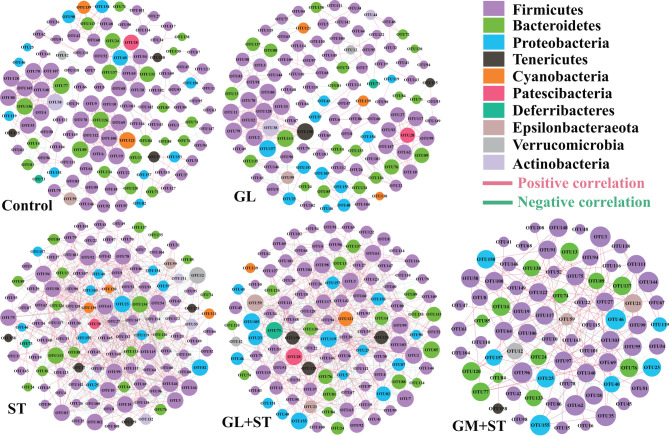
Co-occurrence networks of microbial communities at genus level. A connection stands for a very strong (Spearman's *R* > 0.6) and significant (FDR-adjusted *P* < 0.05) correlation. The size of each node is proportional to the relative abundance; the thickness of each connection between two nodes (edge) is proportional to the value of Spearman's correlation coefficients. Red lines represent significant positive correlations and green lines denote negative correlations.

To determine the co-occurrence patterns of microbes in the different groups, five networks were constructed based on the genus and species levels (Figure 9 and Table 3). Network analysis showed that the values of average degree and graph density in the ST group were higher than those of the other groups (Control, GL, GL + ST, and GM + ST). The modularity values of the co-occurrence networks in all groups were higher than 0.4. The modularity value in the ST group was lower than those in the other groups (Control, GL, GL + ST, and GM + ST). Additionally, negative correlation of the network in the ST group was more than those in the other groups (Control, GL, GL + ST, and GM + ST).

**Table 3:** Topological properties of co-occurrence network.

	**Control**	**GL**	**ST**	**GL + ST**	**GM + ST**
Nodes	120	115	125	114	79
Edges	303	298	428	366	146
Average degree (AD)	5.05	5.183	6.848	6.421	3.696
Graph density (GD)	0.042	0.045	0.055	0.054	0.047
Modularity (MD)	0.893	0.896	0.735	0.877	0.912
Positive correlation	71.95%	67.11%	56.54%	62.57%	64.38%
Negative correlation	28.05%	32.89%	43.46%	37.43%	35.62%

**Figure 10:**
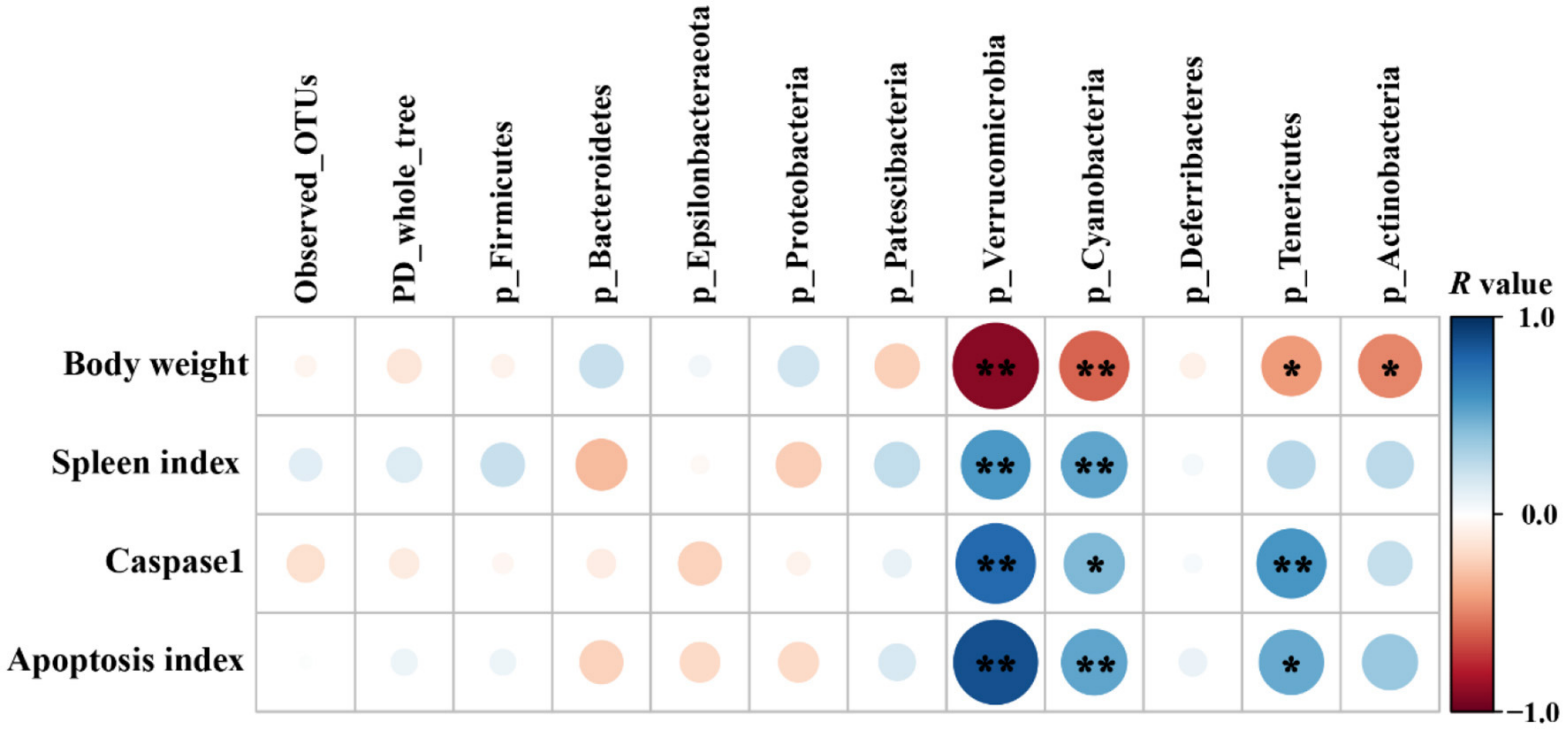
Pearson's correlation analysis between phenotypic variables and the relative abundance of microbial communities (phylum level, *n* = 4/group). The color and dot size represent the correlation coefficient within rows. *P < 0.05; **P < 0.01.

Pearson's correlation coefficient was calculated to reveal correlations between the phenotypic variables and the relative abundance of microbial communities (phylum level) (Figure 10). Correlation analysis showed that the relative abundances of *Verrucomicrobia, Cyanobacteria, Tenericutes*, and *Actinobacteria* were negatively correlated with the final body weight (*P* < 0.05 or *P* < 0.01). The relative abundances of *Verrucomicrobia* and *Cyanobacteria* were positively correlated with the spleen index, caspase-1 activity, and apoptosis index (*P* < 0.05 or *P* < 0.01), while the relative abundance of *Tenericutes* was positively correlated with caspase-1 activity (*P* < 0.01) and apoptosis index (*P* < 0.05).

## Discussion

*Salmonellosis*, caused by food-borne *Salmonella*, is largely associated with tissue injury and intestinal dysbiosis, which accounts for approximately 109.9 million cases and 264,300 deaths globally per year (34). With the strict prohibition of AGP, there is an increasing interest in exploring dietary natural extract products to reduce or prevent against zoonotic enteric pathogen infection and food-borne contamination in industrial livestock production. Previous studies found that GL, an extract from licorice root, could ameliorate HFD-mediated intestinal dysbiosis (20) and *Streptococcus aureus*-induced acute lung injury (25) in murine models. Our previous study found that GL could effectively ameliorate the body weight loss of mice infected with *S*. Typhimurium infection (30), which may be related to the beneficial effects of GL in reducing the inflammation and tissue damage associated with *S*. Typhimurium infection. *Salmonella* Typhimurium may migrate to the liver and spleen *via* the blood circulatory system due to the damage of the intestinal barrier to cause the hepatosplenomegaly, which is characterized by cell apoptosis and inflammatory infiltration (35, 36). In the present study, we found that GL pretreatment significantly prevented *S*. Typhimurium-induced jejunal injury indicated by the higher villous height and splenomegaly indicated by the decreased the spleen index of mice. Additionally, liver injury induced by *S*. Typhimurium infection was also relieved by GL or GM pretreatments as evidenced by the decreased hepatocytic apoptosis and the reduced caspase-1 enzyme activity. GL pretreatment significantly inhibited *S*. Typhimurium colonization in ileum and colon and translocation to spleen and liver (30). In addition, GL enhanced the phagocytosis and bactericidal capacity of BMDM by inducing the JNK/NF-??B signaling pathway-mediated polarization of M1 macrophages that participate in polarized Th1 responses and act as the primary line of defense against intracellular pathogens (18, 37). These results indicate that the attenuated injury of tissues in the GL + ST group may be related to the inhibition of colonization and invasion of *S*. Typhimurium (30), polarization of M1 macrophages (18), improved inflammation response, and intestinal dysbacteriosis.

Inflammatory responses induced by pathogenic infection is one of the most common symptoms of splenomegaly and hepatomegaly (38). The expression of pro-inflammatory cytokines (such as IFN-γ, IL-6, IL-12, and TNF-α) is necessary for the initiation of the innate immune response for pathogen clearance (39). However, excessive pro-inflammatory responses could be extremely harmful to the host (40). Thus, excessive pro-inflammatory responses are tightly balanced by anti-inflammatory cytokines (such as IL-10 and TGF-β) and associated negative feedback loops (41). The ameliorated tissue injury of *S*. Typhimurium-infected mice in GL- or GM-pretreated group mentioned above may be due to the attenuated inflammatory response. Indeed, compared with the uninfected group, *S*. Typhimurium infection significantly induced inflammatory responses as evidenced by the upregulation of pro-inflammatory cytokines (IFN-γ, IL-6, IL-12, and TNF-α) and anti-inflammatory cytokine IL-10 in spleen and liver of mice. Interestingly, GL pretreatment effectively decreased the upregulation of pro-inflammatory cytokines (IFN-γ, TNF-α, and IL-6) induced by ST infection in spleen and liver, and increased secretion of anti-inflammatory cytokine IL-10 in spleen. The results indicate that GL effectively alleviates *S*. Typhimurium-induced inflammatory response by exerting an anti-inflammatory activity.

Intestinal microbes play an important role in maintaining gastrointestinal homeostasis. The intestinal beneficial and commensal microbiota protect host against *Salmonella* colonization *via* competition of adhesion site and nutrition and secretion of metabolite (SCFAs, indole, and bacteriocin) (42, 43). It is reported that *S*. Typhimurium infection is tightly accompanied by intestinal dysbiosis (44, 45). *Salmonella* outcompetes the resident microbes by inducing the host intestinal immune system to secrete reactive oxygen species (ROS) and antimicrobial peptides that are non-specific harmful to the beneficial and commensal microbes (43). In this study, we observed that *Salmonella* infection led to dysbiosis by altering microbial beta diversity and composition. *Salmonella* Typhimurium infection significantly increased the abundances of *Alphaproteobacteria, Verrucomicrobiales*, DTU014, *Prevotellaceae* NK3B31 group, and *Ruminococcaceae* UCG-013, whereas, it decreased the abundances of *Desulfovibrionaceae, Rikenellaceae*, ASF356, *Rikenella, Ruminococcaceae* UCG-009, *Lachnospiraceae* UCG-010, *Alistipes, Odoribacter*, and *Muribaculum*. Knowledge about the altered bacteria caused by *S*. Typhimurium infection remains unclear and needs further investigation. Oral administration of GL significantly increased microbial alpha diversity and altered microbial composition. GL significantly increased the relative abundances of *Oscillibacter, Millionella*, and *Bilophila*, whereas, it decreased the relative abundances of *Verrucomicrobiales*, Family XIII AD3011 group, *Ruminiclostridium* 9, and *Lachnospiraceae* UCG-001. *Oscillibacter* was proposed to exert an anti-inflammatory effect (46). Administration of probiotics increased cecal *Millionella* abundance of mice (47). *Bilophila*, a sulfate-reducing bacterium, is associated with animal-based and fat-enriched diets (48). The intestinal dysbiosis caused by *Salmonella* infection can be reshaped by dietary or antibiotic interventions (31, 49). In the present study, GL or GM pretreatments significantly reshaped the intestinal microbes by reducing microbial alpha diversity, modulating beta diversity, and microbial compositions. The results indicate that the altered microbial diversities and compositions induced by GL pretreatment may be related to the attenuated injury of tissues and pro-inflammatory responses induced by *S*. Typhimurium infection, although, more direct evidences are needed.

Co-occurrence pattern analysis was performed to investigate the microbial interactions, and we found that the values of average degree and graph density of microbial network in the *S*. Typhimurium infected group were higher than those of the other groups (Control, GL, GL + ST, and GM + ST), suggesting that *S*. Typhimurium infection increased the connection among the microbes. All the modularity values of the co-occurrence networks were higher than 0.4, suggesting that these networks had a modular structure (50). The microbial network in the *S*. Typhimurium-infected group was not easier to form a “small world” as evidenced by lower modularity value than those of the other groups (33). Additionally, negative correlation of the microbial networks in the other groups was less than that of the *S*. Typhimurium-infected group, which could be interpreted as a reduction in competitive relationships within intestinal microbes (51). Finally, the correlations between phenotypic variables and microbial communities (alpha diversity and phylum level) were further investigated. The final body weight was obtained from our previous study (30) and reanalyzed with the relative abundance of bacterial phyla. Pearson's correlation analysis showed that the final body weight of mice was negatively correlated with cecal *Verrucomicrobia, Cyanobacteria, Tenericutes*, and *Actinobacteria*. *Verrucomicrobia* and *Cyanobacteria* were positively correlated with the spleen index, caspase-1 activity, and apoptosis index. *Tenericutes* was positively correlated with caspase-1 activity and apoptosis index. The above results indicate that the relieved negative effect caused by *S*. Typhimurium infection may be related to the altered microbial diversities and compositions induced by GL pretreatment.

## Conclusion

Collectively, the present study demonstrates that GL exerts the anti-inflammatory and tissue-protective properties to attenuate ST infection, as indicated by alleviating ST-induced splenomegaly, hepatocytic apoptosis, injury of jejunum and liver, and inflammatory response of liver and spleen in mice. Moreover, we found that GL modulates ST-induced intestinal dysbacteriosis. These findings might give a new perspective into the function of GL in regulating the host immune and intestinal microbiota to defense against pathogen infection. However, further studies about the role of the intestinal microbiota in the progress of GL-mediated anti-*Salmonella* infection are needed.

## ELISA Kit Information

According to the manufacturer's instructions (eBioscience, San Diego, CA), the detailed information about the used ELISA kits is IFN-γ (Assay range: 15.6–1,000 pg/ml; Analytical sensitivity: 1.7 pg/ml; Intra- and Inter-assay CV: <6.8 and <7.4%), IL-12p70 (Assay range: 15.6–1,000 pg/ml; Analytical sensitivity: 10.0 pg/ml; Intra- and Inter-assay CV: 8.3 and 11.0%), TNF-α (Assay range: 31.3–2000 pg/ml; Analytical sensitivity: 3.7 pg/ml; Intra- and Inter-assay CV: 6.5 and 5.7%), IL-6 (Assay range: 31.3–2,000 pg/ml; Analytical sensitivity: 6.5 pg/ml; Intra- and Inter-assay CV: 5 and 8.9%), and IL-10 (Assay range: 39.1–2,500 pg/ml; Analytical sensitivity: 5.28 pg/ml; Intra- and Inter-assay CV: 6.7 and 10.1%).

## Data Availability Statement

Raw sequences have been deposited in the Genome Sequence Archive (GSA) of the BIG Data Center (https://bigd.big.ac.cn/gsa/) under accession number PRJCA004208/CRA003756.

## Ethics Statement

All animal experiments were conducted according to the guidelines and approval of the Institutional Animal Care and Use Committee of Zhejiang University (Permission number: ZJU20160416).

## Author Contributions

YM: conceptualization, supervision, and project administration. BW, XY, and PZ: writing—original draft preparation and writing—review and editing. BW, YZ, and PZ: investigation, methodology, and visualization. All authors have read and agreed to the published version of the manuscript.

## Conflict of Interest

The authors declare that the research was conducted in the absence of any commercial or financial relationships that could be construed as a potential conflict of interest.

## References

[1] LaRock DL Chaudhary A Miller SI. *Salmonellae* interactions with host processes. Nat Rev Microbiol. (2015) 13:191–205. 10.1038/nrmicro3420 25749450 PMC5074537

[2] Haraga A Ohlson MB Miller SI. *Salmonellae* interplay with host cells. Nat Rev Microbiol. (2008) 6:53–66. 10.1038/nrmicro1788 18026123

[3] Liu JS Gu ZN Lu WW Hu DG Zhao X Huang HX. Multiple mechanisms applied by *Lactobacillus* pentosus AT6 to mute the lethal effects of *Salmonella* in a mouse model. Food Funct. (2018) 9:2787–95. 10.1039/C7FO01858D 29691525

[4] Cossart P Sansonetti PJ. Bacterial invasion: the paradigms of enteroinvasive pathogens. Science. (2004) 304:242–8. 10.1126/science.1090124 15073367

[5] Als D Radhakrishnan A Arora P Gaffey MF Campisi S Velummailum R. Global trends in typhoidal *Salmonellosis*: a systematic review. Am J Trop Med Hyg. (2018) 99:10–9. 10.4269/ajtmh.18-0034 30047364 PMC6128363

[6] Kim HB Isaacson RE. *Salmonella* in swine: microbiota interactions. Ann Rev Anim Biosci. (2017) 5:43–63. 10.1146/annurev-animal-022516-022834 27860494

[7] Cox NA Cason JA Richardson LJ. Minimization of *Salmonella* contamination on raw poultry. Annu Rev Food Sci T. (2011) 2:75–95. 10.1146/annurev-food-022510-133715 22129376

[8] Finstad S O'Bryan CA Marcy JA Crandall PG Ricke SC. *Salmonella* and broiler processing in the United States: relationship to foodborne *Salmonellosis*. Food Res Int. (2012) 45:789–94. 10.1016/j.foodres.2011.03.057

[9] Eng SK Pusparajah P Ab Mutalib NS Ser HL Chan KG Lee LH. *Salmonella*: a review on pathogenesis, epidemiology and antibiotic resistance. Front Life Sci. (2015) 8:284–93. 10.1080/21553769.2015.1051243

[10] Broom LJ. The sub-inhibitory theory for antibiotic growth promoters. Poultry Sci. (2017) 96:3104–8. 10.3382/ps/pex114 28595312

[11] Teillant A Brower CH Laxminarayan R. Economics of antibiotic growth promoters in livestock. Annu Rev Resour Econ. (2015) 7:349–74. 10.1146/annurev-resource-100814-125015

[12] China. Announcement of the Ministry of Agriculture and Rural People's Republic of China No. 194. (2019). Available online at: http://www.moa.gov.cn/nybgb/2017/dqq/201801/t20180103_6133925.htm (accessed July 20, 2017).

[13] Tellez G Latorre JD. Editorial: alternatives to antimicrobial growth promoters and their impact in gut microbiota, health and disease. Front Vet Sci. (2017) 4:196. 10.3389/fvets.2017.00196 29177158 PMC5686091

[14] Marshall BM Levy SB. Food animals and antimicrobials: impacts on human health. Clin Microbiol Rev. (2011) 24:718–33. 10.1128/CMR.00002-11 21976606 PMC3194830

[15] Buntyn JO Schmidt TB Nisbet DJ Callaway TR. The role of direct-fed microbials in conventional livestock production. Annu Rev Anim Biosci. (2016) 4:335–55. 10.1146/annurev-animal-022114-111123 26667362

[16] FDA. CFR-Code of Federal Regulations Title 21. Washington, DC: US Food Drug Administration (2017).

[17] Cheng GY Hao HH Xie SY Wang X Dai MH Huang LL. Antibiotic alternatives: the substitution of antibiotics in animal husbandry? Front Microbiol. (2014) 5:217. 10.3389/fmicb.2014.00217 24860564 PMC4026712

[18] Mao YL Wang BK Xu X Du W Li WF Wang YM. Glycyrrhizic acid promotes M1 macrophage polarization in murine bone marrow-derived macrophages associated with the activation of JNK NF-kappa B. Mediat Inflamm. (2015) 2015:372931. 10.1155/2015/372931 PMC4668314 26664149

[19] Matsui S Matsumoto H Sonoda Y Ando K Aizu-Yokota E Sato T. Glycyrrhizin and related compounds down-regulate production of inflammatory chemokines IL-8 and eotaxin 1 in a human lung fibroblast cell line. Int Immunopharmacol. (2004) 4:1633–44. 10.1016/j.intimp.2004.07.023 15454116 PMC7106177

[20] Qiu M Huang KQ Liu YZ Yang YQ Tang HL Liu XX. Modulation of intestinal microbiota by glycyrrhizic acid prevents high-fat diet-enhanced pre-metastatic niche formation and metastasis. Mucosal Immunol. (2019) 12:945–57. 10.1038/s41385-019-0144-6 30755716

[21] Cinatl J Morgenstern B Bauer G Chandra P Rabenau H Doerr HW. Glycyrrhizin, an active component of liquorice roots, and replication of SARS-associated coronavirus. Lancet. (2003) 361:2045–6. 10.1016/S0140-6736(03)13615-X 12814717 PMC7112442

[22] Wang Y Chen Q Shi CX Jiao FZ Gong ZJ. Mechanism of glycyrrhizin on ferroptosis during acute liver failure by inhibiting oxidative stress. Mol Med Rep. (2019) 20:4081–90. 10.3892/mmr.2019.10660 31545489 PMC6797988

[23] Bordbar N Karimi MH Amirghofran Z. The effect of glycyrrhizin on maturation and T cell stimulating activity of dendritic cells. Cell Immunol. (2012) 280:44–9. 10.1016/j.cellimm.2012.11.013 23261828

[24] Itoh K. Augmentation of NK Activity by Several Anti-Inflammatory Agents. 3rd ed. Int Cong Series. Excerpta Medica, Amsterdam (1983). p. 460–4.

[25] Yao L Sun TL. Glycyrrhizin administration ameliorates *Streptococcus* aureus-induced acute lung injury. Int Immunopharmacol. (2019) 70:504–11. 10.1016/j.intimp.2019.02.046 30884430

[26] Carruthers NJ McClellan SA Somayajulu M Pitchaikannu A Bessert D Peng XD. Effects of glycyrrhizin on multi-drug resistant pseudomonas aeruginosa. Pathogens. (2020) 9:776. 10.3390/pathogens9090766 32962036 PMC7557769

[27] Rohinishree YS Negi PS. Effect of licorice extract on cell viability, biofilm formation and exotoxin production by Staphylococcus aureus. J Food Sci Tech Mys. (2016) 53:1092–100. 10.1007/s13197-015-2131-6 27162389 PMC4837708

[28] Naser M Shahab G Mahmood H. Drinking water supplementation of licorice (Glycyrrhiza glabra L. root) extract as an alternative to in-feed antibiotic growth promoter in broiler chickens. GSC Biol Pharm Sci. (2017) 1:20–8. 10.30574/gscbps.2017.1.3.0039

[29] Ibrahim D Sewid AH Arisha AH abd El-fattah AH Abdelaziz AM Al-Jabr OA. Influence of glycyrrhiza glabra extract on growth, gene expression of gut integrity, and campylobacter jejuni colonization in broiler chickens. Front Vet Sci. (2020) 7:612063. 10.3389/fvets.2020.612063 33415133 PMC7782238

[30] Xu XG Gong L Wang BK Wu YP Wang Y Mei XQ. Glycyrrhizin attenuates *Salmonella* enterica serovar Typhimurium infection: new insights into its protective mechanism. Front Immunol. (2018) 9:2321. 10.3389/fimmu.2018.02321 30459751 PMC6232675

[31] Fu A Mo Q Wu Y Wang B Liu R Tang L. Protective effect of *Bacillus* amyloliquefaciens against *Salmonella via* polarizing macrophages to M1 phenotype directly and to M2 depended on microbiota. Food Funct. (2019) 10:7653–66. 10.1039/C9FO01651A 31742290

[32] Jiao S Chen WM Wang JM Zhang L Yang F Lin YB. Plant growth and oil contamination alter the diversity and composition of bacterial communities in agricultural soils across China. Land Degrad Dev. (2018) 29:1660–71. 10.1002/ldr.2932

[33] Jiao S Liu ZS Lin YB Yang J Chen WM Wei GH. Bacterial communities in oil contaminated soils: biogeography and co-occurrence patterns. Soil Biol Biochem. (2016) 98:64–73. 10.1016/j.soilbio.2016.04.005

[34] Stanaway JD Parisi A Sarkar K Blacker BF Reiner RC Hay SI. The global burden of non-typhoidal *Salmonella* invasive disease: a systematic analysis for the Global Burden of Disease Study 2017. Lancet Infect Dis. (2019) 19:1312–24. 10.1016/S1473-3099(19)30418-9 31562022 PMC6892270

[35] Wu JH Hu YJ Du CM Piao JH Yang LC Yang XG. The effect of recombinant human lactoferrin from the milk of transgenic cows on *Salmonella* enterica serovar Typhimurium infection in mice. Food Funct. (2016) 7:308–14. 10.1039/C5FO00817D 26469086

[36] Mathur R Oh H Zhang DK Park SG Seo J Koblansky A. A mouse model of *Salmonella* typhi infection. Cell. (2012) 151:590–602. 10.1016/j.cell.2012.08.042 23101627 PMC3500584

[37] Wang BK Wu YP Liu RR Xu H Mei XQ Shang QQ. Lactobacillus rhamnosus GG promotes M1 polarization in murine bone marrow-derived macrophages by activating TLR2/MyD88/MAPK signaling pathway. Anim Sci J. (2020) 91:e13439. 10.1111/asj.13439 32779289

[38] Ohshio G Furukawa F Fujiwara H Hamashima Y. Hepatomegaly and splenomegaly in Kawasaki disease. Pediatr Pathol. (1985) 4:257–64. 10.3109/15513818509026899 3835550

[39] Lee MS Min YJ. Signaling pathways downstream of pattern-recognition receptors and their cross talk. Annu Rev Biochem. (2007) 76:447–80. 10.1146/annurev.biochem.76.060605.122847 17328678

[40] Kawasaki T Kawai T. Toll-like receptor signaling pathways. Front Immunol. (2014) 5:461. 10.3389/fimmu.2014.00461 25309543 PMC4174766

[41] Wang B Hussain A Zhou Y Zeng Z Wang Q Zou P. Saccharomyces boulardii attenuates inflammatory response induced by *Clostridium* perfringens *via* TLR4/TLR15-MyD8 pathway in HD11 avian macrophages. Poult Sci. (2020) 99:5356–65. 10.1016/j.psj.2020.07.045 33142452 PMC7647824

[42] Caballero S Pamer EG. Microbiota-mediated inflammation and antimicrobial defense in the intestine. Annu Rev Immunol. (2015) 33:227–56. 10.1146/annurev-immunol-032713-120238 25581310 PMC4540477

[43] Gart EV Suchodolski JS Welsh TH Jr Alaniz RC Randel RD. *Salmonella* Typhimurium and multidirectional communication in the gut. Front Microbiol. (2016) 7:1827. 10.3389/fmicb.2016.01827 27920756 PMC5118420

[44] Gillis CC Hughes ER Spiga L Winter MG Zhu W Furtado de Carvalho T. Dysbiosis-associated change in host metabolism generates lactate to support Salmonella growth. Cell Host Microbe. (2018) 23:54–64 e6. 10.1016/j.chom.2017.11.006 29276172 PMC5764812

[45] Khan S Chousalkar KK. *Salmonella* Typhimurium infection disrupts but continuous feeding of *Bacillus* based probiotic restores gut microbiota in infected hens. J Anim Sci Biotechnol. (2020) 11:29. 10.1186/s40104-020-0433-7 32211190 PMC7087389

[46] Li J Sung CY Lee N Ni Y Pihlajamaki J Panagiotou G. Probiotics modulated gut microbiota suppresses hepatocellular carcinoma growth in mice. Proc Natl Acad Sci U S A. (2016) 113:E1306–15. 10.1073/pnas.1518189113 26884164 PMC4780612

[47] Wang F Xu T Zhang Y Zheng T He Y He F. Long-term combined administration of Bifidobacterium bifidum TMC3115 and Lactobacillus plantarum 45 alleviates spatial memory impairment and gut dysbiosis in APP/PS1 mice. FEMS Microbiol Lett. (2020) 367:fnaa048. 10.1093/femsle/fnaa048 32239209

[48] Natividad JM Lamas B Pham HP Michel ML Rainteau D Bridonneau C. Bilophila wadsworthia aggravates high fat diet induced metabolic dysfunctions in mice. Nat Commun. (2018) 9:2802. 10.1038/s41467-018-05249-7 30022049 PMC6052103

[49] Wang X Kong X Qin Y Zhu X Qu D Han J. Milk phospholipid supplementation mediates colonization resistance of mice against *Salmonella* infection in association with modification of gut microbiota. Food Funct. (2020) 11:6078–90. 10.1039/D0FO00883D 32568318

[50] Newman MEJ. Modularity and community structure in networks. P Natl Acad Sci USA. (2006) 103:8577–82. 10.1073/pnas.0601602103 PMC1482622 16723398

[51] Fan KK Weisenhorn P Gilbert JA Shi Y Bai Y Chu HY. Soil pH correlates with the co-occurrence and assemblage process of diazotrophic communities in rhizosphere and bulk soils of wheat fields. Soil Biol Biochem. (2018) 121:185–92. 10.1016/j.soilbio.2018.03.017

